# A Patient With Locally Advanced Mismatch-Repair-Deficient Pancreatic Ductal Adenocarcinoma Successfully Treated With Neoadjuvant Immunotherapy

**DOI:** 10.7759/cureus.14640

**Published:** 2021-04-22

**Authors:** Ronald E Cox, Amit Mahipal, Sakti Chakrabarti

**Affiliations:** 1 Medical Oncology, Medical College of Wisconsin, Milwaukee, USA; 2 Medical Oncology, Mayo Clinic, Rochester, USA; 3 Medical Oncology (GI Oncology), Medical College of Wisconsin, Milwaukee, USA

**Keywords:** pancreatic adenocarcinoma, immunotherapy, ctdna, deficient mismatch repair, liquid biopsy

## Abstract

Pancreatic ductal adenocarcinoma (PDAC) is an aggressive malignancy with a dismal prognosis. Approximately 30% of patients present with locally advanced disease, defined as pancreatic tumor with invasion to adjacent structures, including the vasculatures that preclude an upfront surgical resection. Emerging data suggest that neoadjuvant therapy, typically consisting of systemic chemotherapy followed by concurrent chemoradiation, increases the likelihood of potentially curative R0 resection by downstaging the tumor and improves survival in patients with locally advanced PDAC. PDAC with deficient DNA mismatch repair (dMMR)/microsatellite instability-high molecular signature is exceedingly rare. The role of immunotherapy is emerging in various dMMR gastrointestinal tumors, both in the metastatic and neoadjuvant settings. However, the efficacy of immunotherapy in the neoadjuvant setting in patients with dMMR locally advanced PDAC remains unknown. Herein, we describe a patient who presented with unresectable dMMR locally advanced PDAC and underwent neoadjuvant immunotherapy with pembrolizumab that resulted in a remarkable reduction of the tumor size, rendering the tumor resectable. Furthermore, the presence of dMMR signature in the tumor was detected by circulating tumor DNA analysis. This is the first report, to our knowledge, of the successful use of neoadjuvant immunotherapy in a patient with locally advanced PDAC.

## Introduction

Pancreatic ductal adenocarcinoma (PDAC) is a lethal malignancy characterized by nonspecific symptoms, diagnosis in an advanced stage, and a dismal prognosis [1]. More than half of the patients with PDAC present with metastatic disease [2]. Approximately 30% of patients present with locally advanced disease, defined as a pancreatic tumor with invasion into adjacent structures, including the vasculatures that preclude an upfront surgical resection [2]. Administration of chemotherapy and radiation therapy before surgery, popularly known as neoadjuvant therapy (NAT), is emerging as a promising therapeutic strategy for such patients that often renders the tumor resectable [3]. The potential advantages of NAT over upfront surgery followed by adjuvant therapy include early treatment of micrometastatic disease potentially reducing the risk of systemic relapse, increased probability of curative margin-negative resection, greater likelihood of delivery of adequate systemic therapy that could be delayed or omitted because of postoperative complications, and an opportunity to exclude tumors with highly aggressive biology where surgery is unlikely to be beneficial. Currently, the National Comprehensive Cancer Network guideline for the treatment of pancreatic cancer includes NAT as an acceptable treatment strategy for locally advanced PDAC [4].

Recent literature reporting the remarkable success of immunotherapy in tumors with microsatellite instability-high (MSI-H)/deficient DNA mismatch repair (dMMR) signature has generated considerable interest in this tumor type [5]. Pembrolizumab, an approved immunotherapeutic agent that works by blocking programmed death 1 (PD-1) receptor on T lymphocytes, has shown impressive anti-cancer activity in a wide variety of dMMR tumors, including dMMR gastrointestinal (GI) tumors [5-7]. Clinical trials reporting remarkable shrinkage of metastatic dMMR tumors with immunotherapy [5,6,8] provide a rationale for utilizing immunotherapy for tumor downstaging before surgery in various GI cancers. In this case report, we describe the successful use of immunotherapy in a patient with dMMR unresectable locally advanced PDAC who became amenable to surgical resection after considerable tumor shrinkage following treatment with pembrolizumab. A written consent from the patient was obtained before submission of this case report.

## Case presentation

**Figure 1:**
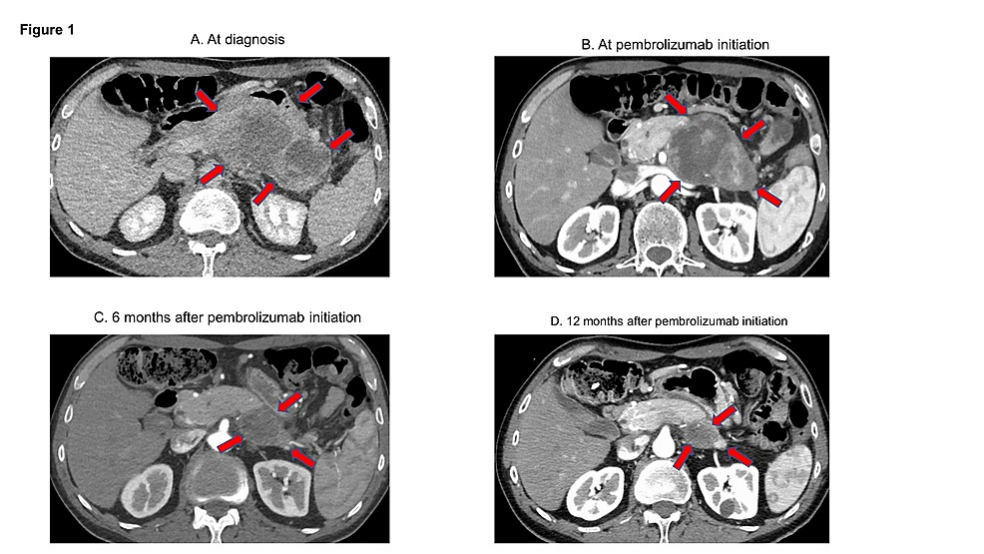
Representative CT images of pancreatic mass at diagnosis and after treatment (A) At diagnosis, the pancreatic body and tail were occupied by a large heterogeneous solid and cystic mass measuring 11.6 x 6.4 cm. Invasion into the surrounding vasculature was noted. (B) About three months into the initial treatment (hyperthermia with low-dose chemotherapy), the pancreatic mass size decreased, measuring 7.9 x 5.9 cm. Pembrolizumab was started at this time. (C) The pancreatic mass demonstrated further shrinkage measuring 5.3 x 3.7 cm, about six months after the first dose of pembrolizumab.  (D) Approximately 12 months after the first dose of pembrolizumab, the pancreatic tumor showed markedly decreased size, measuring 3.1 x 3.0 cm.

A 56-year-old man presented with progressive upper abdominal pain, diminished appetite, weight loss, and fatigue. The examination was unremarkable without any palpable abdominal mass, ascites, or any other significant physical findings, except long-standing psoriatic rash. He was prescribed omeprazole, which did not alleviate his symptoms. Consequently, a series of tests were prescribed, including complete blood count, comprehensive metabolic panel, esophagogastroduodenoscopy (EGD), colonoscopy, and CT scan of chest, abdomen, and pelvis. Blood tests were unremarkable with normal liver function tests. EGD and colonoscopy were unremarkable. CT scan revealed a large heterogeneous mixed solid and cystic mass replacing the pancreatic body and tail measuring 11.6 x 6.4 cm (Figure 1A) with invasion into the surrounding vasculature without any distant metastatic disease. The CA 19-9 level was elevated at 6232 units/ml (normal range, 0-35). The patient underwent evaluation with endoscopic ultrasound, which demonstrated a large mass at the pancreatic body and tail with a normal left lobe of the liver and no regional lymphadenopathy. The tissue obtained by fine-needle aspiration (FNA) confirmed poorly differentiated adenocarcinoma. His overall activity level was marginally limited suggesting an Eastern Cooperative Oncology Group (ECOG) performance status of 1 at presentation. The patient was evaluated by a medical oncologist who recommended neoadjuvant chemotherapy with modified FOLFIRINOX (5-fluorouracil, irinotecan, and oxaliplatin) as the tumor was unresectable at presentation. However, the patient was highly concerned about the potential adverse reactions of the chemotherapy and chose to pursue an unconventional form of treatment consisting of hyperthermia combined with low-dose chemotherapy, details of which are unavailable. He received this therapy for about three months with poor tolerability. After this treatment (three months from diagnosis), a CT scan showed a modest decrease in the pancreatic mass size, measuring 7.6 x 5.9 cm, without any evidence of distant metastasis (Figure 1B).

At this time, he was referred to a surgeon to assess the resectability of the tumor. His tumor was classified as borderline resectable due to arterial abutment. Additionally, the patient was a Jehovah's Witness, which presented another barrier to surgery as he was unwilling to receive any blood products. The surgeon recommended continued NAT.

The patient was hesitant to receive any chemotherapy because of his previous poor tolerance. A tumor genomic profiling was considered to explore targeted therapy options, but the FNA specimen did not have adequate tissue for the test. As a result, a circulating tumor DNA (ctDNA)-based tumor genomic profiling (Guardant360 CDx) was performed, which revealed an MSI-H/dMMR tumor without any other actionable alterations, providing an opportunity for utilizing immunotherapy. Notably, the patient also had a long-standing history of psoriasis controlled with topical agents. After a detailed discussion of the potential benefits versus risks with immunotherapy, including the risk of psoriasis exacerbation, the patient consented to proceed with immunotherapy. The treatment with pembrolizumab was initiated at the dose of 200 mg intravenously every three weeks. He developed several grade 3 immune-related adverse events (irAEs) after two doses of pembrolizumab, including psoriatic skin rash, arthralgia, and fatigue. Pembrolizumab was discontinued and the patient was treated with a topical and systemic steroid to the resolution of those symptoms. The option of treatment with chemoradiation followed by surgery was presented, but the patient was unwilling to follow this path. A mutual plan of close observation with serial imaging studies and serum CA 19-9 levels was made after a lengthy discussion. A germline mutation testing at this time confirmed a diagnosis of Lynch syndrome.

**Figure 2:**
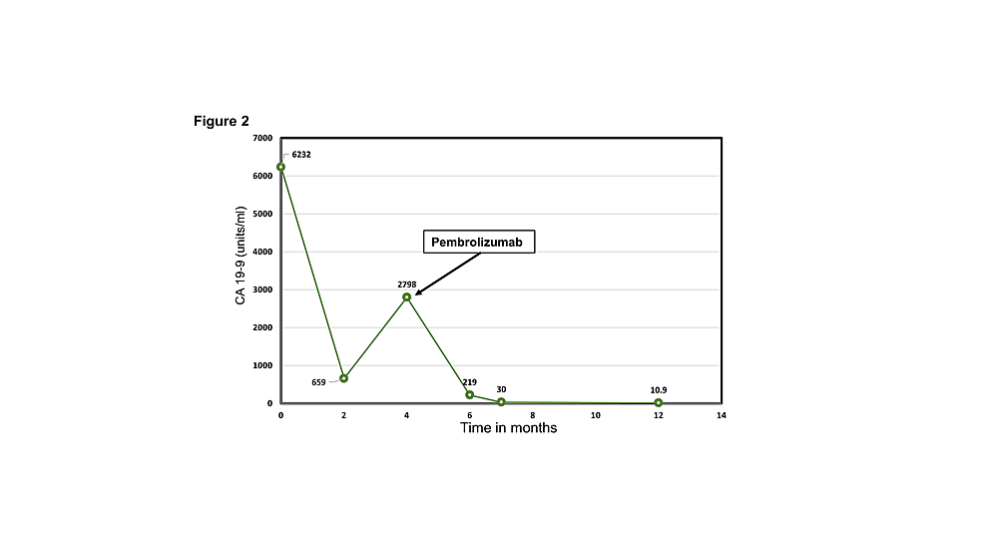
Serial CA 19-9 levels demonstrating normalization after the treatment with pembrolizumab

A CT scan performed approximately six months after pembrolizumab initiation showed a remarkable response with the tumor measuring 3.6 x 3.9 cm (Figure 1C). At this time, the tumor was deemed resectable, but the patient was unwilling to pursue a surgical resection due to his concern regarding post-surgical complications. Serial measurements of serum CA 19-9 levels showed a steady decline with normalization of the level about four months after the first dose of pembrolizumab (Figure 2).

A major concern at this point was the regrowth of the tumor since he had not received any cancer-directed therapy for several months. In the interim period, he fully recovered from the adverse effects of pembrolizumab. We presented the option of re-treatment with pembrolizumab after a detailed discussion of the risks. He chose to receive pembrolizumab, which was resumed along with a small dose of oral prednisone (10 mg daily). He tolerated two more cycles of pembrolizumab before experiencing a significant worsening of the psoriatic rash. Pembrolizumab was stopped. At this time, two options were discussed with the patient - 1. the option of surgery, and 2. concurrent chemoradiation followed by surgery or observation (rationale discussed in the following section). The patient chose observation. He is currently being observed with serial imaging studies and serum CA 19-9 level measurements. At his latest evaluation, approximately a year after his first treatment with pembrolizumab (Figure 1D), the tumor size was 3.1 x 3.0 cm. The CA 19-9 level remained within the normal range (Figure 2).

## Discussion

We describe a patient with dMMR unresectable locally advanced PDAC who received neoadjuvant immunotherapy with pembrolizumab that rendered the tumor resectable. The quintessential challenge in treating patients with locally advanced PDAC is achieving the adequate tumor response necessary to ensure resection with negative surgical margins that enhances the likelihood of long-term survival. Emerging data suggest that NAT, which typically consists of systemic chemotherapy followed by concurrent chemotherapy and radiation, downstages the tumor, helps achieve R0 resection, and improves disease-free and overall survival [9-11]. Among the neoadjuvant chemotherapy regimens, modified FOLFIRINOX (short-term infusional 5-fluorouracil, leucovorin, irinotecan, and oxaliplatin) has gained popularity for patients with a good ECOG performance status [11]. The other commonly utilized neoadjuvant chemotherapy regimen consists of gemcitabine plus nab-paclitaxel [12]. In this instance, although modified FOLFIRINOX was recommended as the initial NAT, the patient chose to receive an unconventional treatment regimen (hyperthermia and low-dose chemotherapy) that did not render the tumor resectable.

The key finding in our patient was the presence of a dMMR signature in the tumor, which is exceedingly rare in patients with PDAC, present only in 1%-2% of patients [13]. A plethora of clinical trials have demonstrated remarkable anti-tumor activity of immunotherapy irrespective of the organ or tissue of origin [5,6,8], leading to an accelerated tissue- or site-agnostic approval of pembrolizumab by the Food and Drug Administration (FDA) for adult and pediatric patients with unresectable or metastatic MSI-H or dMMR solid tumors resistant to standard therapies. The immunotherapeutic drugs used most extensively in the current clinical practice belong to the broad category of immune checkpoint inhibitors (ICIs) that include anti-PD-1 agents (e.g., pembrolizumab, nivolumab), anti-PD-L1 (programmed death-ligand 1) agents (e.g., atezolizumab), and cytotoxic T-lymphocyte-associated protein 4 (CTLA-4) blocking agents (e.g., ipilimumab). Among the GI tumors, the efficacy of single-agent pembrolizumab has been evaluated in patients with dMMR metastatic colorectal cancer (mCRC) in the first-line setting in a recently reported trial (KEYNOTE-177) [6]. In this trial, treatment-naive patients with mCRC were randomized to either standard doublet chemotherapy or immunotherapy with single-agent pembrolizumab (study arm) [6]. The trial showed that pembrolizumab was superior to chemotherapy with respect to progression-free survival (median, 16.5 vs. 8.2 months; hazard ratio, 0.60; P = 0.0002) and overall response rate 43.8% vs. 33.1%. Furthermore, treatment with pembrolizumab resulted in a complete radiological response in 11% of the patients. FDA has recently approved pembrolizumab as a first-line therapy for dMMR mCRC based on the KEYNOTE-177 trial data.

The preliminary data with immunotherapy utilized in the neoadjuvant setting for the dMMR GI tumors, such as colon cancer [14] and gastric cancer [15], are encouraging. Chalabi et al. reported the result of the NICHE study [14] in which patients with early-stage dMMR colon cancer received neoadjuvant immunotherapy with combined PD-1 (nivolumab) and CTLA-4 blockade (ipilimumab) followed by surgery. The response to the neoadjuvant immunotherapy was remarkable, with 12 complete pathological responses (60%) and 19 major pathological responses (≤10% viable residual tumor) among the 20 treated patients. These data motivated us to treat the patient with pembrolizumab for tumor downstaging. The patient indeed showed an impressive tumor response, as described in the previous section.

Although genotyping solid tumors to inform targeted therapy selection has been widely accepted, comprehensive profiling is not feasible in 25%-50% of patients because of the inadequate amount of tumor tissue in the biopsy [16]. Consequently, analysis of ctDNA to identify actionable targets has emerged as a viable alternative, a strategy supported by multiple studies [17,18]. The lack of adequate tissue in the current patient's FNA specimen led us to use the Guardant360 CDx, an FDA-approved commercially available ctDNA assay for genomic profiling that revealed the dMMR signature in the tumor helping us to choose immunotherapy for tumor downstaging.

Since ICIs trigger irAEs, patients with pre-existing autoimmune disorders (psoriasis in the current case) are at increased risk of high-grade irAEs. However, retrospective studies have reported similar side-effect profiles and comparable efficacy with ICIs in patients with pre-existing autoimmune disorders [19] that led us to recommend pembrolizumab for our patient. The other challenge we faced was the re-introduction of ICI after he developed grade 3 irAEs. The decision to consider re-introduction of pembrolizumab for this patient was driven by several factors, including a lack of alternative treatment, impressive tumor shrinkage after only two doses of pembrolizumab, absence of potentially life-threatening irAEs, and retrospective studies confirming the feasibility of ICI re-introduction in such scenarios [20]. Although our patient developed irAEs again with the re-introduction of pembrolizumab, irAEs were manageable.

A unique feature of the dMMR tumors treated with ICI is that pathologic complete response is often noted in the resected specimens despite the presence of residual tumor on preoperative imaging studies, a phenomenon demonstrated in the patients with dMMR mCRC. We discussed this phenomenon with the patient and explained that surgery might reveal a complete pathologic response. Since the patient declined surgery, we recommended concurrent chemoradiation followed by observation as one of the options.

## Conclusions

The current case report suggests that tissue-agnostic anti-tumor activity of ICIs can potentially be exploited successfully as NAT in dMMR locally advanced PDAC, and hence testing the tissue for dMMR signature should be considered. Furthermore, the presence of an autoimmune disorder should not be an absolute contraindication to immunotherapy, especially if treatment options are limited and the autoimmune disorder is manageable.

In routine clinical practice, occasionally biopsy specimens may not contain enough tissue for genomic studies. In such cases, ctDNA-based genomic profiling is a reasonable alternative that might provide an important therapeutic target, as highlighted by the current case report.
